# Generalized problematic internet use and perfectionism in portuguese university students

**DOI:** 10.1192/j.eurpsy.2021.1186

**Published:** 2021-08-13

**Authors:** B. Rodrigues Maia, M.J. Soares, A.T. Pereira, M. Marques, F. Carvalho, A. Macedo

**Affiliations:** 1 Faculty Of Philosophy And Social Sciences, Centre For Philosophical And Humanistic Studies, Portugal, Universidade Católica Portuguesa, Braga, Portugal; 2 Institute Of Psychological Medicine, Faculty of Medicine, University of Coimbra, Coimbra, Portugal; 3 Institute Of Psychological Medicine, Faculty Of Medicine, University of Coimbra, coimbra, Portugal; 4 Coimbra Hospital And University Centre, Portugal, Coimbra Hospital and University Centre, Coimbra, Portugal; 5 Espaço Psicológico – Consultório De Psicologia, Coimbra, Portugal, Espaço Psicológico – Consultório de Psicologia, Coimbra, Portugal, Coimbra, Portugal; 6 Institute Of Psychological Medicine, Faculty Of Medicine, University of Coimbra, Coimbra, Portugal

**Keywords:** Problematic Internet use, Perfectionism, University Students

## Abstract

**Introduction:**

Although there are several empirical studies exploring the relationship between problematic Internet use and personality traits, few had considered perfectionism.

**Objectives:**

To explore the association between generalized problematic Internet use and perfectionism.

**Methods:**

A sample of 433 Portuguese university students (M_age_ = 20.15 years, SD = 1.77, range = 18-25 years) completed the Generalized Problematic Internet Use Scale 2/GPIU and the Multidimensional Perfectionism Composite Scale – short version.

**Results:**

GPIU total score (rs=.16**), Mood Regulation (rs=.22**), and Deficient Self-Regulation (rs=.13**) were correlated with Positive Striving factor. GPIU total score (rs=.38**), Preference for Online Social Interaction (rs=.16**), Mood Regulation (rs=.28**), Deficient Self-Regulation (rs = .33**), and Negative Consequences (rs=.41**) were significantly correlated with Evaluative Concerns factor. A one-way between groups analysis of variance was conducted to explore the relation between GPIU and perfectionism. Subjects were divided into three groups according to their GPIU risk levels (Group1:low-risk; Group2:medium-risk; Group3:high-risk). There was a statistically difference at p >.05 level in Positive Striving scores for the three risk level groups: F (2,430)=4.39, p=.013, and in Evaluative Concerns scores, F (2,430)=28.83, p=<.001. Post-hoc comparisons using the Tukey USD test, for Positive Striving, indicated that the mean score for Group1 (M=39.21, SD=8.56) was significantly different from Group3 (M=43.69, SD=9.74). Considering Evaluative Concerns, the mean score for Group1 (M=39.86, SD=11.31) was significantly different from Group2 (M=46.91, SD=11.42) and from Group3 (M=51.75, SD=8.54).

**Conclusions:**

GPIU is consistently related to maladaptive perfectionism. Future longitudinal studies are needed to clarify the bidirectional association between GPIU and perfectionism traits.

